# Greenhouse gas emissions from sub-tropical agricultural soils after addition of organic by-products

**DOI:** 10.1186/2193-1801-3-491

**Published:** 2014-08-30

**Authors:** Dai H Nguyen, Johannes Biala, Peter R Grace, Clemens Scheer, David W Rowlings

**Affiliations:** Institute for Future Environments, Queensland University of Technology, Brisbane, QLD 4000 Australia; The Organic Force, Wynnum, QLD 4178 Australia; Sugarcane Research Institute, Binh Duong, Vietnam

**Keywords:** Carbon dioxide, Greenhouse gas emissions, Organic soil amendments, Nitrous oxide

## Abstract

As the cost of mineral fertilisers increases globally, organic soil amendments (OAs) from agricultural sources are increasingly being used as substitutes for nitrogen. However, the impact of OAs on the production of greenhouse gases (CO_2_ and N_2_O) is not well understood. A 60-day laboratory incubation experiment was conducted to investigate the impacts of applying OAs (equivalent to 296 kg N ha^-1^ on average) on N_2_O and CO_2_ emissions and soil properties of clay and sandy loam soils from sugar cane production. The experiment included 6 treatments, one being an un-amended (UN) control with addition of five OAs being raw mill mud (MM), composted mill mud (CM), high N compost (HC), rice husk biochar (RB), and raw mill mud plus rice husk biochar (MB). These OAs were incubated at 60, 75 and 90% water-filled pore space (WFPS) at 25°C with urea (equivalent to 200 kg N ha^-1^) added to the soils thirty days after the incubation commenced. Results showed WFPS did not influence CO_2_ emissions over the 60 days but the magnitude of emissions as a proportion of C applied was RB < CM < MB < HC < MM. Nitrous oxide emissions were significantly less in the clay soil compared to the sandy loam at all WFPS, and could be ranked RB < MB < MM < CM < UN < HC. These results led to linear models being developed to predict CO_2_ and N_2_O emissions as a function of the dry matter and C/N ratio of the OAs, WFPS, and the soil CEC. Application of RB reduced N_2_O emissions by as much as 42-64% depending on WFPS. The reductions in both CO_2_ and N_2_O emissions after application of RB were due to a reduced bioavailability of C and not immobilisation of N. These findings show that the effect of OAs on soil GHG emissions can vary substantially depending on their chemical properties. OAs with a high availability of labile C and N can lead to elevated emissions of CO_2_ and N_2_O, while rice husk biochar showed potential in reducing overall soil GHG emissions.

## Introduction

Carbon dioxide (CO_2_) and nitrous oxide (N_2_O) are two of the most important greenhouse gases (GHGs) that contribute to global warming (IPCC 2007). Nitrous oxide also contributes to stratospheric ozone layer depletion (Ravishankara et al. 2009). Nitrous oxide has a global warming potential 298 times greater than CO_2_ over a hundred year time horizon and a long lifetime of 114 years (Forster et al. 2007; IPCC 2007). Globally, it has been estimated that N_2_O emissions have increased from 11 Tg N_2_O-N yr^-1^ in the early 17^th^ century to 17.7 Tg N_2_O-N yr^-1^ in 1994 (Kroeze et al. 1999) and that the concentration of N_2_O in the atmosphere is increasing by 0.8% annually (IPCC 1994). The concentration of CO_2_ has also increased from 280 ppm in pre-industrial times to 392 ppm in the early 21^st^ century (Tans 2012), highlighting that a reduction in the concentration of both gases would aid in the mitigation of climate change.

Nitrous oxide is produced from nitrification and denitrification processes (Davidson et al. 1986) and are influenced by pH (Law et al. 2011), soil moisture, and availability of C and N substrates (Beare et al. 2009). Both processes of nitrification and denitrification can occur simultaneously in a soil at aerobic and anaerobic micro-sites at soil water contents between 60% and 80% water-filled pore space (WFPS), with an increase in denitrification when WFPS exceeds 80% (Davidson et al. 1986; Dalal et al. 2003; Bolan et al. 2004). Furthermore, N_2_O emissions can be stimulated by the application of N fertiliser and organic soil amendments (Aulakh et al. 1984; Eichner 1990; Wang et al. 2011, 2012). Decomposition of organic materials generates labile C compounds (Chatterjee et al. 2008) which enhances the denitrification rate in soils (Robertson and Tiedje 1988; Wallenstein et al. 2006; Pérez et al. 2010).

The increasing cost of mineral fertilisers and the degradation of native soil fertility in production systems are placing greater emphasis on the use of low cost organic by-products for both carbon and nutrient management (Zhao et al*.*^2009^) both as a sole source of N or in combination with mineral fertilisers. A wide range of organic residues from intensive animal production, food and fibre processing and municipal/commercial sources are available for use as organic soil amendments (OAs). The application of OAs to soil supplies macro- and micro-nutrients, improves soil chemical and physical properties and promotes plant growth (Quilty and Cattle 2011). Organic compounds, including biochar, are negatively charged because of the phenolic and carboxyl groups in their structure (Verheijen et al. 2009; Widowati et al. 2011) and increase surface negative charge (Naidu and Syers 1992) that may lead to increased cation exchange capacity (CEC) (Chan et al. 2008; Peng et al. 2011; Widowati et al. 2011) and improve soil fertility. The applications of these OAs, including manures and biochar have also been shown to increase pH to more favourable levels for plant growth in acidic soils (Naidu and Syers 1992; Whalen et al. 2000; Amiri and Fallahi 2009; Yuan et al. 2011).

OAs can be raw or processed (e.g. dried, pelletised, composted, digested, pyrolyzed) and display markedly different characteristics with respect to nutrient status and availability. For example, rice husk as the main by-product of rice mills represents 22% of the total weight (Umamaheswaran and Batra 2008), and is generated at an annual rate of nearly 164 million tonnes worldwide considering there is 745 million tonnes of rice produced globally (FAO-STAT 2014). Whilst part of the rice husk is used for heating, much of it is discarded. Production of rice husk biochar may offer a pathway for the beneficial use of the rice husk in agricultural soils (e.g. Masulili et al. 2010), and in turn reduce GHG emissions (Zhang et al. 2010, 2012; Wang et al. 2012). Wang et al. ( 2011) found that application of 50 t ha^-1^ rice husk biochar to paddy soils decreased N_2_O emissions by 73.1%.

Mill mud is a sugar milling by-product, with approximately 60 million tonnes generated worldwide on an annual basis of which 2 million tonnes is generated in Australia (Barry et al. 2000). Mill mud is recognised as a valuable agricultural OA containing lime and a range of nutrients which improves soil conditions (Kumar et al*.*^1985^) and crop yields (Yaduvanshi and Yadav 1999). Mill mud is applied as an alternative to synthetic N fertilisers for sugarcane production, however application rates in excess of 150 t ha^-1^are considered to be well above crop requirements with soils showing elevated levels of nutrients and heavy metals after repeated applications (Barry et al. 2000). Composted mill mud (0.5 to 1.48% total N) (Chapman 1996), has neutral pH and low phyto-toxicity, also making it suitable for agricultural use (Meunchang et al. 2005).

While the rice husk and the mill mud OAs are relatively low in N (total and available) food processing residues exist with elevated N levels, (e.g. gelatine manufacturing residues). These by-products are highly putrescible and classified as regulated waste and have to be processed before they can be applied to agricultural soils. Composted residues from the manufacture of gelatine also represent a valuable OA with relatively high N content (1.7% to 3.2%) (Biala and Smeal 2008).

Whilst the agronomic benefits of OAs have been the focus of many studies (Zebarth et al. 1999; Speir et al. 2004; Zhao et al. 2009) there is little complementary information on the contribution of OAs to GHG emissions and global warming, specifically the production of N_2_O, and its relationship to the mineralisation of OAs. The mineralisation of OAs applied to soil is dependent on the C:N ratio of the OA and soil physical and chemical conditions, including soil moisture content. For example, the suppression of N_2_O emissions on the application of biochar may be due to increased soil pH and changed soil aeration (Cavigelli and Robertson 2001). Plant residues with a low C:N ratio normally induce relatively high N_2_O emissions (Huang et al. 2004) when soils remain aerobic, but soil N_2_O production is depressed when soil conditions become anaerobic (Li et al. 2013b). In contrast, when inorganic sources of N are available, N_2_O emissions under anaerobic conditions are orders of magnitude higher than under aerobic conditions (Linn and Doran 1984).

The primary objective of this study was to evaluate the impact of applying biochar and OAs (with equivalent bulk and/or N loadings) relevant to the sugar cane industry on the emissions of two major GHGs (CO_2_ and N_2_O) from soils with similar agronomic history and fertility but contrasting textures. Laboratory incubations (under controlled conditions) provide valuable information on the mineralisation of OAs and the production of GHGs which may be masked in field situations under variable temperature, moisture and soil conditions. Laboratory incubations also provide a quantitative means of pre-selecting OAs for more extensive field testing before being used for commercial purposes. Changes in soil pH and CEC were also assessed in an attempt to derive empirical relationships describing CO_2_ and N_2_O evolution over a range of soil water contents for predictive modelling purposes. The experimental design also included observations when soils were amended with OAs alone and in combination with mineral N fertiliser, a common practice in cropping systems.

## Materials and methods

A 60-day laboratory incubation experiment was used to evaluate the influence of soil texture and moisture on the production of N_2_O and CO_2_ after the application of OAs and mineral N at rates relevant to the sugar cane industry. Two contrasting soils (sandy loam and clay) were incubated at 25°C with 18 treatments (6 OAs × 3 soil moisture levels) with five replicates. The treatments were (i) an un-amended (UN) control; the application of the equivalent of (ii) 110 t ha^-1^ raw mill mud (MM); (iii) 60 t ha^-1^ rice husk biochar (RB); (iv) 60 t ha^-1^ raw mill mud plus 25 t ha^-1^ RB (MB); (v) 110 t ha^-1^ composted mill mud (CM); and (vi) 23 t ha^-1^ high N compost (HC). After 30 days, mineral N fertiliser (equivalent to 200 kg N ha^-1^) was added to all treatments.

### Organic soil amendments

The RB was produced from rice husks by thermal pyrolysis at 350 to 500°C and supplied by Barmac Industries Pty Ltd. MM and CM were provided by the Broadwater Sugar Mill. MM was stockpiled for several weeks prior to use, while the CM also included bagasse, and was aged for at least eight months following a three month windrow composting process. MM and CM were manually screened through a 4 mm stainless mesh for removing bulky fragments before application. The HC was made from gelatine manufacturing residues, which are blended with shredded vegetation residues. The mixture, which was composted for 4–5 weeks, contained approximately 30% (v/v) wastewater sludge and skutch (boiled and minced cattle hide) and about 70% (v/v) bulking materials including shredded vegetation residues, old compost and boiler ash. The HC was not a fully composted and stabilised compost product and was mechanically screened to <20 mm mesh at the composting operation. The chemical and physical properties of OAs are listed in Table 1.

**Table 1:** **The characteristics of organic amendments added to sugarcane soils (sandy loam and clay) from Broadwater (NSW)**

	Mill mud (MM)	Composted mill mud (CM)	High N Compost (HC)	Rice husk biochar (RB)	MM + RB (MB)
pH (1:5 H_2_O)	7.2	6.9	8.0	9.0	ND
Dry matter (kg)	37,070	54,230	16,123	55,920	43,520
CEC (cmol^(+)^ kg^-1^)	18.8	15.5	ND^*^	5.6	ND
Total C (g kg^-1^)	250	120	280	464	365
Labile C (g kg^-1^)	1.4	0.1	5.8	0	1.0
Total N (g N kg^-1^)	8.9	5.8	21.0	4.0	6.3
Mineral N (mg N kg^-1^)	0	5.5	4891	0	0
C/N ratio	28.1	20.7	13.3	116.0	57.7
Moisture (%)	66.3	50.7	29.9	6.8	48.8
Total C applied (kg C ha^-1^)	9,268	6,508	4,514	25,947	15,866
Total N applied (kg N ha^-1^)	330	315	339	224	273
BD^¶^ - sandy loam (g cm^–3^)	1.05	1.13	1.17	1.02	1.05
BD^¶^ - clay (g cm^–3^)	0.92	0.97	1.07	0.92	0.89

*ND: not determined. MB, combined MM and RB. ^¶^soil bulk density after mixing the OAs in the soils.

The magnitude of soil based GHG emissions after application of OAs is a function of both the quantity and quality (C and N contents) of the OAs and the moisture content of the soil during the incubation. HC was applied at a rate equivalent to the total N loading of MM. RB was applied at a bulk rate slightly higher than previously reported for tropical cropping systems (Zhang et al. 2010; Haefele et al. 2011) to ensure the N addition was comparable to the other OAs. MB was a combination of MM and RB with the same N loading as CM.

### Description of soil sampling and preparation

Both soils (sandy loam and clay) were collected from a sub-tropical sugarcane plantation in Broadwater, northeast NSW, Australia (29°00′S 153°25′E). Bulk soil was collected from a depth of 0–20 cm across a uniformly managed field, then air-dried, sieved through a 2 mm stainless mesh, and stored at 4°C until used. Soil properties are described in Nguyen et al. ( 2013). Briefly, the sandy loam and the clay soil have clay contents of 12.0 and 49.7%, total organic C contents of 2.2 and 2.5%, and soil bulk density of 1.2 and 1.17 g cm^-3^, respectively. Total N contents (0.2%) and pH levels (4.9) were identical for both soils.

### Soil incubation

OAs were thoroughly mixed with 200 cm^3^ of soil (240 g sandy loam soil or 234 g clay soil, oven-dry weight base). The soil was packed into a 15 cm high PVC cylindrical pipe with 5.05 cm inner diameter to achieve the target bulk density (Table 1). Soil cores were pre-incubated for seven days at 25°C at 60% WFPS after addition of deionised water. WFPS was calculated as:


where:

- GWC: gravimetric water content (g g^-1^)
- BD: soil bulk density (g cm^-3^)
- 2.65 assumes the soil particle density (g cm^-3^)

After pre-incubation, soil moisture was adjusted to 60%, 75%, and 90% WFPS for respective treatments to cover the full range from aerobic to anaerobic soil conditions (Weier et al. 1993). Each core was placed in a 1-litre glass jar (hereafter called "chamber"), which could be capped with a rubber septum lid. The lids were removed after each gas sampling event to ensure the gases in the chamber returned to atmospheric concentrations prior to recommencing the incubation. The cores were maintained at a consistent temperature of 25°C in an incubator. Constant soil moisture was maintained by weighing the cores every three days with the addition of deionised water if required. The incubation experiment was conducted in two stages, each of which lasted a total of 30 days. At the beginning of the second stage (Day 30) mineral N fertiliser (200 kg N ha^-1^ as dissolved urea) was added to all treatments to replicate a common field practice of mixing organic and inorganic fertilisers.

### Measurements and calculations of N_2_O and CO_2_ emissions

Flux rates of N_2_O and CO_2_ were determined at 1, 2, 3, 5, 7, 10, 12, 15, 20, 25, and 30 days after the start of incubation and again (using the same gas sampling frequency) after the addition of N fertiliser. Gas samples were taken from chamber headspace at closure of the septum lid and three hours after closure (Nguyen et al. 2014) by inserting a syringe through the rubber septum. Gas samples were immediately transferred from syringe into evacuated Exetainer tubes (Labco Ltd, Buckinghamshire, UK) for storage until analysis.

Headspace concentrations were measured by gas chromatography using a Shimadzu GC-2014, equipped with an electron capture detector (ECD) for N_2_O and a thermal conductivity detector (TCD) for CO_2_. Flux rates (F) of N_2_O and CO_2_ were calculated using the following equation:


where, b: Increase in headspace concentration (ppb min^-1^); A_CH_: Basal area of the measuring chamber (m^2^); MW is either MW_N2O-N_: Molecular weight of N_2_O-N (28 g mol^-1^) or MW_CO2-C_: Molecular weight of CO_2_-C (12 g mol^-1^); MV_corr._: Temperature corrected molecular volume (m^3^ mol^-1^); V_CH_: Volume of the measuring headspace chamber (m^3^); 60: conversion from minutes to hours; 10^6^: Converts g to μg; 10^9^ converts ppb to μL m^-3^.


where: MV_corr._: is defined as above; 0.02241: 22.41 L mol volume (m^3^ mol^-1^); T: Air temperature during the measurement (°C).

To convert [μg m^-2^ h^-1^] to [g or kg ha^-1^ d^-1^] the following formulae were used:


where, 24 converts hours to days, 10^4^ converts m^2^ to hectares, and 10^6^ and 10^9^ convert μg to g and μg to kg, respectively.

### Soil analysis

The concentrations of exchangeable NH_4_^+^-N and NO_3_^-^-N, CEC, and pH were measured after 60 days of incubation. NH_4_^+^-N and NO_3_^-^-N were determined after shaking 20 g of fresh wet soil with 100 mL 2 M KCl for 2 h and the filtered extracts analysed by AQ2^+^ SEAL Analytical WI, USA (Carter and Gregorich 2008). CEC of each soil was determined by ICP-OES (Inductively Coupled Plasma – Optical Emissions Spectroscopy) after 2 g of air-dried soil was extracted with 40 mL 1 M ammonium chloride (buffer at pH 7). Soil pH_(H2O)_ was determined on a 1:5 soil:solution basis at room temperature using a pH meter.

Labile C concentrations in OAs were determined by a methodology adapted from the method described in AS4454-2003 (Australian Standard 2003). A 100 g sample was extracted in 150 mL hot deionised water (100°C) while the sample was mixed in a tumbler at 30 rpm for 30 minutes. After extraction, the sample was filtered and analysed for labile C via a total organic C analyser.

### Statistical analysis

Three-way analysis of variance (ANOVA) was used for determining the influence of OAs, soil texture and WFPS on CO_2_ and N_2_O over the first 30 days, and the second 30 days after addition of the equivalent to 200 kg N as urea. Three-way ANOVAs were also used to determine the impact of OAs, soil texture and WFPS on CEC, pH after and mineral N (NH_4_^+^ and NO_3_^-^) after 60 days. The N_2_O emissions, NH_4_^+^ and pH results were log transformed to meet conditions of normality and homogeneity of variances. We also attempted to develop a predictive linear model of CO_2_ and N_2_O production for the two soils in response to WFPS over the two 30 day time periods by including the specific properties of the OAs (i.e. the C/N ratio and total dry matter addition) as independent variables. Statistical analyses were performed using Mathematica ( 2013).

## Results

### CEC and soil pH

There was no effect of WFPS on CEC after 60 days for all OA treatments in both soils, however highly significant texture and treatment effects were apparent (P < 0.001) (Table 2). In the sandy loam, CEC increased (on average) by 2.3 cmol^(+)^ kg^-1^ or 80% compared to UN, whilst in the clay soil CEC increased by 1.4 cmol^(+)^ kg^-1^ or 9.7%. In terms of CEC, the only OAs that were significantly different to the unamended soil were HC and CM in the sandy loam.

**Table 2:** **CEC in two sugarcane soils (sandy loam and clay) from Broadwater (NSW) at Day 60 of incubation at three WFPS levels following addition of OAs**

	CEC (cmol ^(+)^kg ^-1^soil)					
	Sandy loam			Clay		
	WFPS (%)			WFPS (%)		
Treatment	60	75	90	60	75	90
UN	2.9 ± 0.2	2.9 ± 0.1	2.8 ± 0.2	14.6 ± 1.2	14.4 ± 1.1	14.2 ± 1.0
MM	4.6 ± 0.2	5.2 ± 0.4	5.1 ± 0.3	15.7 ± 1.4	15.5 ± 1.3	16.0 ± 1.0
RB	4.2 ± 0.2	4.1 ± 0.3	3.9 ± 0.11	14.7 ± 1.0	14.7 ± 1.0	14.9 ± 1.1
MB	4.5 ± 0.2	4.6 ± 0.1	4.7 ± 0.1	15.7 ± 1.1	15.6 ± 1.3	15.6 ± 1.3
CM	5.1 ± 0.4	5.3 ± 0.2	5.3 ± 0.1	16.2 ± 1.3	15.6 ± 1.1	16.4 ± 1.2
HC	6.9 ± 0.8	7.2 ± 0.3	7.1 ± 0.2	16.7 ± 0.9	17.2 ± 1.2	16.6 ± 0.9

Urea was added on Day 30. Values present the mean with SE (n = 4).

There was no effect of WFPS or texture on pH after 60 days for the OA treatments in both soils (Table 3), however a treatment effect was apparent (P < 0.01), with a 0.5 pH unit increase after 60 days in the RB treatment in the sandy loam soil being the most significant.

**Table 3:** **pH in two sugarcane soils (sandy loam and clay) from Broadwater (NSW) at Day 60 of incubation at three WFPS levels following addition of OAs**

	pH (H _2_O)					
	Sandy loam			Clay		
	WFPS (%)			WFPS (%)		
Treatment	60	75	90	60	75	90
UN	4.05 ± 0.12	3.93 ± 0.18	4.20 ± 0.22	4.51 ± 0.10	4.14 ± 0.20	4.21 ± 0.16
MM	4.23 ± 0.14	4.15 ± 0.13	4.25 ± 0.21	4.56 ± 0.12	4.41 ± 0.15	4.19 ± 0.18
RB	4.49 ± 0.06	4.41 ± 0.06	4.48 ± 0.05	4.51 ± 0.28	4.37 ± 0.15	4.30 ± 0.09
MB	4.35 ± 0.09	4.39 ± 0.12	4.41 ± 0.16	4.42 ± 0.12	4.21 ± 0.10	4.27 ± 0.08
CM	4.21 ± 0.14	4.18 ± 0.17	4.54 ± 0.11	4.20 ± 0.18	4.16 ± 0.15	4.20 ± 0.16
HC	4.16 ± 0.12	3.96 ± 0.16	4.27 ± 0.13	4.09 ± 0.16	4.05 ± 0.17	4.11 ± 0.15

Urea was added on Day 30. Values present the mean with SE (n = 4).

### Daily flux rates of CO_2_ and N_2_O emissions

**Figure 1:**
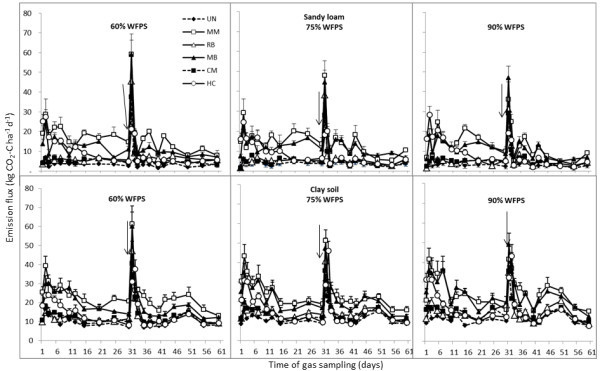
**CO**_**2**_ **emissions following addition of organic soil amendments to two sugarcane soils (sandy loam and clay) from Broadwater (NSW) monitored over a 60-day incubation period.** Urea (200 kg N ha^-1^) was added on day 30. Arrows indicate the time of N addition and vertical bars present the SE (n = 5).

Daily flux rates of CO_2_ from the sandy loam and clay soils over the 60 days of the incubation are presented in Figure 1. Increasing WFPS did not significantly influence CO_2_ emissions. During the first 30 days, the highest CO_2_ flux rates were measured on the second day of the incubation (average 14.7 kg C ha^-1^ and 26 kg C ha^-1^ from the sandy loam and clay soil respectively). The lowest flux rates were measured in UN (4.3 kg C ha^-1^ and 16.3 kg C ha^-1^ from the sandy loam and clay soil respectively) and the highest rates in the MM treatments (25.1 kg C ha^-1^ and 41.5 kg C ha^-1^ respectively). On day 2, the CO_2_ flux rates from the RB and CM amended treatments were only marginally higher than the UN (on average 5.3 kg C ha^-1^ and 17.8 kg C ha^-1^ from the sandy loam and clay soil respectively). By day 30, CO_2_ flux rates averaged 6.9 kg C ha^-1^ and 13.3 kg C ha^-1^ from the sandy-loam and clay soil respectively with the MM soil still the highest emitter of all the OAs (12.7 and 18.5 kg C ha^-1^ respectively). After 30 days, the RB and CM soils still produced relatively low CO_2_ emissions, comparable to the UN control soils.

In terms of changes in the magnitude of CO_2_ flux over the 30 days, the HC amended soils initially produced relatively high daily CO_2_ emissions (average 27.7 kg C ha^-1^) with little difference between soil types; however by day 30 the CO_2_ emissions in HC had decreased to 7.8 kg C ha^-1^. The MB amended soils produced relatively high daily CO_2_ emissions at day 2 (average 27.5 kg C ha^-1^) declining to 13.5 kg C ha^-1^ by day 30.

After addition of urea (equivalent to 85.7 kg C ha^-1^) on day 30, the highest daily flux rates of CO_2_ for the entire incubation period of 60 days were recorded on day 31 (average 36 and 41 kg C ha^-1^ for the sandy-loam and clay soil respectively). The majority of the CO_2_ from the urea application was emitted over the next 2–3 days, with all treatments (in both soils) stabilising to pre-urea addition levels after day 33. The daily CO_2_ flux rates from days 33–60 were relatively stable across all treatments (average 6.2 and 14.5 kg C ha^-1^ from the sandy loam and clay soil respectively), with the highest emissions from the MM and MB amended treatments, on average 10.1 and 19.7 kg C ha^-1^ for the sandy loam and clay soil respectively. Daily flux rates from the other OAs (HC, RB and CM) in both soil types were similar to UN and approximately one-half of the flux rates recorded for MM and MB.

**Figure 2:**
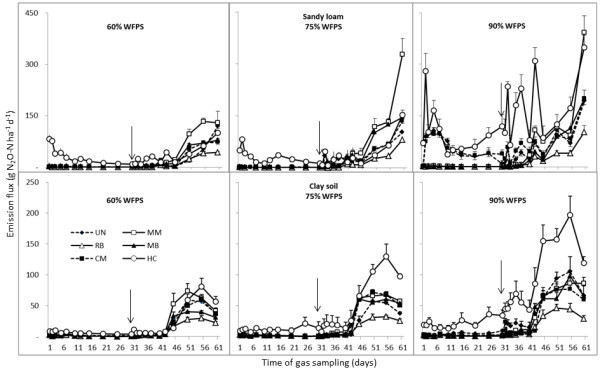
**N**_**2**_ **O emissions following addition of organic soil amendments to two sugarcane soils (sandy loam and clay) from Broadwater (NSW) monitored over a 60-day incubation period.** Urea (200 kg N ha^-1^) was added on day 30. Arrows indicate the time of N addition and vertical bars present the SE (n = 5).

Daily flux rates of N_2_O from the sandy loam and clay soils over 60 days are presented in Figure 2. Changes in WFPS had a significant impact on N_2_O emissions, in particular the unamended sandy loam soil where at 90% WFPS, the average daily N_2_O emissions over the first 10 days were 77 g N ha^-1^ compared to 1–2 g N ha^-1^ from the lower WFPS treatments. During the first 30 days, the average daily N_2_O flux rate from the MM, RB and MB treatments in both soils (regardless of WFPS) was only 1 g N ha^-1^. The average daily N_2_O flux rates from the CM treatment were also similar to these treatments but in the clay soil only. The HC amended soil produced the highest N_2_O emissions of all the OAs, with average daily N_2_O emissions over the first 30 days of 56 and 13 g N ha^-1^ from the sandy loam and clay soil respectively.

In both soils, the daily N_2_O emissions gradually increased after the addition of urea (equivalent to 200 kg N ha^-1^). The highest daily N_2_O emissions from the sandy loam soil were measured at day 60 (average 162 g N ha^-1^), ranging from 75 g N ha^-1^ (RB) to 283 g N ha^-1^ (HC). The highest daily N_2_O emissions from the clay soil were measured at day 55 (average 75 g N ha^-1^) ranging from 35 g N ha^-1^ (RB) to 136 g N ha^-1^ from the HC amended soil. The average daily N_2_O emissions from days 31–60 were 45 g N ha^-1^ and 28 g N ha^-1^ from the sandy loam and clay soil respectively. Daily N_2_O emissions ranged from 17 g N ha^-1^ (RB) to 83 g N ha^-1^ (HC) in the sandy loam and from 13 g N ha^-1^ (RB) to 55 g N ha^-1^ (HC) in the clay soil with mid-range emissions from the UN control.

### Cumulative emissions of CO_2_ and N_2_O

Both soil type and the type of OA had highly significant influences (P < 0.001) on total CO_2_ emissions over 30 and 60 days (Table 4). After 30 days, the CO_2_ emissions from the UN clay soil (304 kg C ha^-1^) (averaged across WFPS) were nearly 3 times the magnitude of those measured from the sandy loam soil. Total CO_2_ emissions from the OA amended soils after 30 days ranged from 155–488 kg C ha^-1^ in the sandy loam soil and 384–699 kg C ha^-1^ in the clay soil with the lowest emissions from the RB and CM treatments and the highest from the MM amended soils. Background CO_2_ emissions from unamended soil after 60 days were 230 kg C ha^-1^ and 666 kg C ha^-1^ for the sandy loam and clay soil respectively. Total CO_2_ emissions from the OA amended soils after 60 days ranged from 309–818 kg C ha^-1^ in the sandy loam soil and from 782–1339 kg C ha^-1^ in the clay soil, with the lowest emissions from the RB and CM treatments, and the highest emissions from MM amended soils. In terms of CO_2_ emissions after both 30 and 60 days, the treatments could be ranked UN < RB = CM < HC < MB < MM with the CO_2_ emissions from the first 30 days representing just over one-half of the CO_2_ emissions after 60 days (including CO_2_ emissions from urea).

**Table 4:** **Total CO**_**2**_ **emitted and proportion of applied C lost as CO**_**2**_ **after addition of OAs to two sugarcane soils (sandy loam and clay) from Broadwater (NSW) after 30 and 60 days incubation**

		Applied C (t ha ^-1^)	Sandy loam soil				Clay soil			
Treatment			Total CO _2_–C emissions		Net CO _2_-C ^‡^/C applied		Total CO _2_–C emissions		Net CO _2_-C ^‡^/C applied	
			(kg CO _2_–C ha ^-1^)		(%)		(kg CO _2_–C ha ^-1^)		(%)	
			D30	D60	D30	D60	D30	D60	D30	D60
60% WFPS	UN	0	101 ± 6	235 ± 16	-	-	279 ± 11	612 ± 22	-	-
	MM	9.27	533 ± 36	910 ± 46	4.7 ± 0.4	7.3 ± 0.5	686 ± 26	1314 ± 85	4.4 ± 0.4	7.6 ± 0.9
	RB	25.95	174 ± 9	395 ± 17	0.3 ± 0.03	0.6 ± 0.1	346 ± 7	724±13	0.3 ± 0.1	0.4 ± 0.1
	MB	15.87	364 ± 17	661 ± 10	1.7 ± 0.1	2.7 ± 0.1	590 ± 15	1084 ± 38	2.0 ± 0.2	3.0 ± 0.3
	CM	6.50	158 ± 9	312 ± 17	0.9 ± 0.2	1.2 ± 0.4	344 ± 14	721 ± 28	1.0 ± 0.2	1.7 ± 0.5
	HC	4.51	298 ± 22	487 ± 19	4.4 ± 0.6	5.6 ± 0.6	401 ± 27	762 ± 40	2.7 ± 0.5	3.3 ± 0.9
75% WFPS	UN	0	116 ± 5	254 ± 18	-	-	306 ± 10	659 ± 15	-	-
	MM	9.27	497 ± 71	820 ± 97	4.1 ± 0.7	6.1 ± 1.1	713 ± 45	1331 ± 75	4.4 ± 0.4	7.2 ± 0.7
	RB	25.95	156 ± 8	332 ± 24	0.2 ± 0.04	0.3 ± 0.1	419 ± 12	860 ± 27	0.4 ± 0.02	0.8 ± 0.1
	MB	15.87	397 ± 31	718 ± 31	1.8 ± 0.2	2.9 ± 0.3	624 ± 18	1179 ± 30	2.0 ± 0.1	3.3 ± 0.2
	CM	6.50	153 ± 12	299 ± 19	0.6 ± 0.2	0.7 ± 0.3	383 ± 15	786 ± 31	1.2 ± 0.3	2.0 ± 0.3
	HC	4.51	286 ± 12	458 ± 15	3.8 ± 0.3	4.5 ± 0.6	438 ± 14	838 ± 32	2.9 ± 0.4	3.9 ± 0.5
90% WFPS	UN	0	93 ± 3	203 ± 3	-	-	329 ± 11	728 ± 22	-	-
	MM	9.27	433 ± 43	725 ± 49	3.7 ± 0.4	5.6 ± 0.5	698 ± 28	1373 ± 44	4.0 ± 0.3	7.0 ± 0.7
	RB	25.95	136 ± 11	302 ± 27	0.2 ± 0.04	0.4 ± 0.1	448 ± 5	891 ± 21	0.5 ± 0.1	0.6 ± 0.1
	MB	15.87	370 ± 7	635 ± 28	1.7 ± 0.05	2.7 ± 0.2	698 ± 25	1324 ± 67	2.3 ± 0.2	3.8 ± 0.5
	CM	6.50	154 ± 13	317 ± 26	0.9 ± 0.2	1.8 ± 0.4	424 ± 16	838 ± 29	1.5 ± 0.3	1.7 ± 0.5
	HC	4.51	300 ± 30	459 ± 32	4.6 ± 0.7	5.7 ± 0.8	471 ± 10	885 ± 34	3.1 ± 0.4	3.5 ± 1.1

^‡^Net CO_2_ emissions after subtracting unamended losses and CO_2_ from urea (85.7 kg C ha^-1^) in the case of D30-60.

Urea (200 kg N ha^-1^) was added at Day 30. Values are mean with SE (n = 5).

There were significant differences between OAs with respect to the proportion of the applied C loss as CO_2_ after 30 and 60 days (Table 4). When averaged across WFPS levels, the amount of C evolved as CO_2_ (corrected for both background and urea based emission) and expressed as a percentage of total C applied was similar for both soils, ranging from 0.3-4.3% after 30 days, and 0.5-6.7% after 60 days. In terms of CO_2_ production (as a proportion of C applied), the OAs could be ranked RB < CM < MB < HC < MM, similar to the ranking of absolute CO_2_ emissions.

Linear models were developed to estimate total CO_2_ (kg C ha^-1^) production from the soils in response to adding OAs and WFPS over the two 30 day time periods. The models took into account the addition of urea at 30 days.


Where CN is the C/N ratio of the OA, DM is dry matter applied (kg ha^-1^) at day 0, CEC is cation exchange capacity of the soil (cmol^(+)^ kg^-1^) at day 0 and WFPS is water filled pore space (%).

All three variables (soil type, OA and WFPS) had highly significant influences (P < 0.001) on total N_2_O emissions over 30 and 60 days (Table 5). The average total N_2_O emissions after 30 days were 1.37 and 0.42 kg N ha^-1^ from the sandy loam and clay soil respectively. After 60 days, the average total N_2_O emissions were 2.36 and 1.36 kg N ha^-1^ from the sandy loam and clay soil respectively. After 30 days, the N_2_O emissions from all OAs (except HC) in the sandy loam were comparable to UN (average 33 g N ha^-1^) at 60% and 75% WFPS. In contrast, the HC soils had emitted (on average) 0.69 kg N ha^-1^ at 60% and 75% levels. At 90% WFPS, N_2_O emissions for RB, MM and MB treatments in the sandy loam remained low, but the N_2_O emissions from the UN and CM treatments increased thirty-fold (to 1.44 kg N ha^-1^) over the first 30 days when compared to the two lower WFPS levels. Emissions from the HC treatment at 90% WFPS increased four-fold to 2.7 kg N ha^-1^.

**Table 5:** **Total N**_**2**_ **O emissions from two sugarcane soils (sandy loam and clay) from Broadwater (NSW) after periods of 30 and 60 days incubation at three WFPS levels following addition of OAs**

	Cumulative N _2_O emission (kg N _2_O-N ha ^-1^)					
	Sandy loam soil			Clay soil		
	WFPS			WFPS		
Treatment	60%	75%	90%	60%	75%	90%
			Day 30			
UN	0.04 ± 0.01	0.02 ± 0.002	1.40 ± 0.08	0.06 ± 0.01	0.04 ± 0.01	0.16 ± 0.02
MM	0.02 ± 0.001	0.02 ± 0.003	0.05 ± 0.003	0.03 ± 0.01	0.02 ± 0.002	0.04 ± 0.01
RB	0.02 ± 0.001	0.02 ± 0.003	0.04 ± 0.01	0.03 ± 0.01	0.05 ± 0.02	0.04 ± 0.01
MB	0.02 ± 0.001	0.02 ± 0.003	0.02 ± 0.001	0.04 ± 0.01	0.03 ± 0.002	0.03 ± 0.01
CM	0.02 ± 0.001	0.03 ± 0.01	1.48 ± 0.15	0.03 ± 0.004	0.04 ± 0.01	0.07 ± 0.03
HC	0.65 ± 0.11	0.73 ± 0.09	2.73 ± 0.38	0.16 ± 0.02	0.38 ± 0.13	0.72 ± 0.19
			Day 60			
UN	1.07 ± 0.07	1.49 ± 0.26	3.73 ± 0.24	0.97 ± 0.07	0.89 ± 0.06	1.93 ± 0.27
MM	1.97 ± 0.21	3.20 ± 0.33	3.65 ± 0.15	1.11 ± 0.13	1.26 ± 0.15	1.63 ± 0.14
RB	0.61 ± 0.03	0.81 ± 0.07	1.14 ± 0.17	0.51 ± 0.03	0.60 ± 0.05	0.81 ± 0.14
MB	1.17 ± 0.16	2.09 ± 0.25	2.25 ± 0.24	0.72 ± 0.03	1.12 ± 0.13	1.51 ± 0.14
CM	1.08 ± 0.04	1.49 ± 0.17	4.06 ± 0.45	0.86 ± 0.08	1.20 ± 0.05	1.42 ± 0.06
HC	1.99 ± 0.12	2.32 ± 0.44	8.29 ± 0.85	1.27 ± 0.15	2.46 ± 0.39	4.25 ± 0.52

Urea (200 kg N ha^-1^) was added on Day 30. Values present the mean with SE (n = 5).

In the clay soil, when considering both OAs and WFPS, the trend in N_2_O production over the first 30 days was similar to the sandy loam, however in the clay soil the increase in N_2_O emissions at 90% WFPS were not as pronounced as in the sandy loam. Emissions from the UN treatment in the clay soil were (on average) 49 g N ha^-1^ at 60% and 75% WFPS but only increased three-fold at 90% WFPS. Total N_2_O emissions from RB, MM and MB were relatively consistent across the three WFPS levels (average of 35 g N ha^-1^). The highest emissions were found from HC, increasing stepwise from 162–716 g N ha^-1^ as WFPS increased from 60-90%.

Considering both soil types, the impact of adding RB (when compared to UN) on N_2_O emissions during the first 30 days was a reduction of 1.36 and 0.11 kg N ha^-1^ for the sandy loam and clay soil respectively. Similar reductions in N_2_O were found in the MM and MB treatments.

After the addition of urea (200 kg N ha^-1^) at day 30, total N_2_O emissions over the next 30 days (averaged over all WFPS levels) increased nearly five-fold in the sandy soil (from 0.41 to 1.95 kg N ha^-1^) and over ten-fold in the clay soil (from 0.11 to 1.25 kg N ha^-1^) compared to the first 30 days. In the sandy loam, N_2_O emissions increased from 1.0 to 2.33 kg N ha^-1^ in the UN treatment as WFPS increased from 60 to 90% WFPS and from 1.95 to 3.6 kg N ha^-1^ in MM. The largest increase in N_2_O emissions was found in the HC treatment (1.34 to 5.56 kg N ha^-1^) when WFPS increased from 60 to 90%. Total N_2_O emissions over the second 30 days in RB were only 0.59 kg N ha^-1^ at 60% WFPS, increasing to 1.1 kg N ha^-1^ at 90% WFPS, the latter only marginally higher than the emissions from the UN treatments at 60% WFPS.

In the clay soil, the trend in N_2_O production (considering both OAs and WFPS) over the second 30 days was similar to that found in the sandy loam, however the increases in N_2_O emissions were not as pronounced as those found in the sandy loam at 90% WFPS. The latter observation was also found during the first 30 days.

Considering both sandy loam and clay soils, the overall impact of adding RB (when compared to UN) on N_2_O emissions during the second 30 days was a relatively consistent average reduction of 1.12 kg N ha^-1^. In the sandy loam, when compared to UN, the MB amendment also provided a small reduction in N_2_O (103 g N ha^-1^), whilst CM increased N_2_O production by 243 g N ha^-1^. There were large increases in N_2_O emissions (compared to UN) from the MM and HC treatments in the sandy loam during the second 30 days (1.27 and 3.22 kg N respectively). In the clay soil, the MM, MB and CM treatments provided an average reduction in N_2_O emissions of 306 kg N ha^-1^, whilst HC increased N_2_O production by 1.76 kg N ha^-1^. In terms of N_2_O production over both 30 and 60 days, the OAs could be ranked RB < MB < MM < CM < UN < HC. The impact of applying RB on N_2_O emissions was consistent across both soil types. During the second 30 day period (after the addition of urea) the N_2_O emissions declined (compared to UN) on average by 42% at the two lower WFPS and 64% at the high WFPS.

Linear models were developed to estimate total N_2_O (g N ha^-1^) production from the soils in response to adding OAs and WFPS over the two 30 day time periods. The models took into account the addition of urea at 30 days.


### Variation in mineral N during the incubation

After 60 days, the concentrations of mineral N (measured as NH_4_^+^ and NO_3_^-^) were significantly higher (P < 0.001) in the clay (average 276 mg N kg^-1^) compared to the sandy loam (average 176 mg N kg^-1^) across all WFPS (Table 6). The average mineral N content of all the OAs was approximately the same as the UN treatment in the sandy loam, but 40 mg N kg^-1^ higher than UN in the clay soil. The mineral N content of the HC amended soil after 60 days was found to be significantly higher (P < 0.01) compared to all the other OAs (including UN), mainly due to an increase in soil NO_3_^-^ (average difference of 85 mg N kg^-1^). The addition of RB also resulted in significant higher NO_3_^-^ levels after 60 days (average 45 mg N kg^-1^) compared to UN.

**Table 6:** **Mineral N levels in two sugarcane soils (sandy loam and clay) from Broadwater (NSW) after 60 days and following the application of OAs (Day 0) and urea** ^**‡**^ **(Day 30)**

		Sandy loam ^§^		Clay ^§^	
Treatment		NH _4_^+^-N NO _3_^-^-N		NH _4_^+^-N NO _3_^-^-N	
		mg kg^-1^ soil			
60% WFPS	UN	82 ± 22	109 ± 12	81 ± 12	106 ± 13
	MM	45 ± 9	109 ± 18	78 ± 10	112 ± 41
	RB	45 ± 1	155 ± 26	48 ± 8	141 ± 15
	MB	39 ± 8	115 ± 4	87 ± 18	154 ± 9
	CM	38 ± 7	99 ± 16	54 ± 10	164 ± 24
	HC	47 ± 4	210 ± 4	78 ± 26	206 ± 39
75% WFPS	UN	86 ± 17	136 ± 15	93 ± 4	179 ± 3
	MM	67 ± 21	190 ± 40	99 ± 6	155 ± 6
	RB	43 ± 14	151 ± 23	125 ± 32	239 ± 29
	MB	47 ± 11	161 ± 22	89 ± 14	172 ± 7
	CM	39 ± 9	137 ± 7	67 ± 14	188 ± 13
	HC	56 ± 4	213 ± 24	87 ± 9	259 ± 5
90% WFPS	UN	39 ± 8	73 ± 22	91 ± 14	178 ± 28
	MM	25 ± 5	69 ± 18	94 ± 25	205 ± 26
	RB	12 ± 4	135 ± 42	117 ± 14	227 ± 24
	MB	17 ± 3	97 ± 22	99 ± 27	149 ± 17
	CM	28 ± 5	67 ± 14	96 ± 20	230 ± 23
	HC	26 ± 5	172 ± 20	78 ± 16	351 ± 52

Values are mean with SE (n = 4).

^‡^the equivalent of 200 kg ha^-1^ of N.

^§^prior to OAs being added, NH_4_^+^-N was 19 and 19 mg kg^-1^ and NO_3_^-^-N was 19 and 17 mg kg^-1^ in sandy loam and clay soil, respectively.

## Discussion

### Influence of CEC and pH on N_2_O emissions

As the cost of mineral fertilisers continue to increase, organic residues are increasingly being utilised for their dual role in improving soil health, improving chemical properties (such as CEC and pH) and nutrient supply. Our study only found significant increases in CEC after the addition of the two OAs which had relatively low C/N ratios (HC and CM), the latter at a relatively high loading rate and CEC. Whilst there are studies that report the benefits of RB in terms of improving soil CEC (Liang et al*.*^2006^), a distinction must be made between short and long-term applications of RB and OAs generally. Our results are consistent with those of Whalen et al. ( 2000) who assessed a range of animal manures and Masulili et al. ( 2010) who found no difference in CEC after application of rice husk biochar. Typically, repeated application of OAs is required to change soil properties (Shiralipour et al*.*^1992^). Whilst Whalen et al. ( 2000) reported changes in pH after single (but relatively high loadings of manure), in our study, a significant change in pH was only observed after the addition of highly alkaline RB (pH 9). Our results are consistent with Masulili et al. ( 2010) who suggest that rice husk biochar could be considered as a substitute for lime materials to increase the pH of acidic soils. In contrast, the pH of HC amended clay soil declined due to the relatively high sulphur (ca. 1.5%) content of HC (Biala and Smeal 2008).

### Influence of OA types on CO_2_ and N_2_O emissions

Changes in WFPS did not influence the magnitude of CO_2_ emissions from the sandy loam or clay soils over 60 days, which is consistent with the incubation study of Ruser et al. ( 2006), and field studies of Frank et al. ( 2002) and Drewitt et al. ( 2002). Even though the unamended soils were similar in terms of initial soil organic C content, the fact the clay soil respired 3 times as much CO_2_ indicates very different partitioning of the native soil organic matter pools and availability of more decomposable forms of soil organic matter in the clay. On addition of the OAs, we observed the highest fluxes over the first 2 days, which has also been reported by Alotaibi and Schoenau ( 2013). On application of N fertiliser at day 30, CO_2_ emissions were enhanced by the hydrolysis of the urea (van Zwieten et al*.*^2010^), similar to reports by Li et al. ( 2013a) after applying supplemental fertilisers.

All OAs promoted higher CO_2_ emissions over the 60 days (compared to the unamended soil), however the fact we fixed the rate of total N application of OAs means CO_2_ emissions are directly linked to the proportion of decomposable organic C within a specific OA (Ajwa and Tabatabai 1994). Whilst there was considerable difference in the amount of DM added (equivalent to 16–56 t DM ha^-1^), the total amount of OA applied in combination with the C/N ratio generally explains the behaviour of the individual OAs in terms of both absolute and the proportional CO_2_ emissions except where composting has been part of the preparation of the OA process resulting in the labile C and mineral N fractions of the OA being low (e.g. CM). Significantly lower rates of C mineralisation have also been reported by Hartz et al. ( 2000) when comparing composts to manure amendments. Our linear models (combining DM, C/N, WFPS and soil CEC) predicted the CO_2_ emissions from CM in comparison to other OAs (including the composted HC), but the HC had sufficiently high levels of labile C (as an easily degradable C source) and mineral N (for assimilation by microorganisms in the decomposition process). Nitrogen immobilisation takes place in soils amended with raw organic material (such as raw mill mud) (Negro et al. 1999) and high C-to-N ratio organic amendments (Ambus et al. 2001; Craine et al. 2007). Our results are consistent with the fact that OAs with a C/N ratio in excess of 40 have insufficient N to meet microbial demands for rapid decomposition (Virgil and Kissel 1991) and initially degrade at a slower rate than those with lower C/N ratios. Whilst the MB treatments (C/N = 57) did not conform to this observation of reduced CO_2_ emissions this is due to the fact there was a clear partitioning between readily decomposable and resistant C fractions due to the mixing of MM and RB feedstocks, and the C/N ratio of the blended product did not adequately characterise the product in this respect.

Our observation of significant reduced CO_2_ emissions with the addition of biochar compared to other OAs is consistent with Singh et al. ( 2010) and Zimmerman et al. ( 2011) but increases in CO_2_ emissions after biochar addition have also been reported by Rogovska et al. ( 2011) and Zheng et al. ( 2012). Biochar decreases the bioavailability of soluble organic substrates due to absorption on their surfaces suppressing CO_2_ emissions (van Zwieten et al. 2009) however where increases in CO_2_ emissions have been reported after the addition of biochar, this is normally accompanied by a reduction in extractable NO_3_^-^, or immobilisation, to support microbial growth and decomposition (Ippolito et al. 2012; Zheng et al. 2012). In our study, extractable NO_3_^-^ levels were significantly higher in the RB treated soils than the unamended after 60 days, supporting the observation of Major et al. ( 2010) and Singh et al. ( 2012) that the predominately aromatic structure of high C/N biochars have little bioavailable C for inducing immobilisation.

The availability of N from OAs and fertiliser sources determines the rate of both nitrification and denitrification and subsequent N_2_O production from soils (Andersen and Petersen 2009). Nitrous oxide emissions produced over the 60 days in both soils were negatively correlated with the C/N ratio of the OA (i.e. higher N_2_O emissions at lower C/N), similar to the observations of Huang et al. ( 2004) and Chen et al. ( 2013). The highest emissions were consistently observed from the HC amended soils at all WFPS, with high levels of readily available (mineral) N and labile C to promote denitrification at the highest WFPS (Robertson and Tiedje 1988; Regina et al. 1998; Mohn et al. 2000; Garcia-Montiel et al. 2003; Bateman and Baggs 2005; Wallenstein et al. 2006; Pérez et al. 2010). The significant increase in N_2_O emissions we observed once WFPS exceeded 75% has been demonstrated in many studies (Weier et al. 1993; Rudaz et al. 1999; Ruser et al. 2006). The increased N_2_O emissions observed after N fertilisation are also similar to the observations of Uchida et al. ( 2013), Ji et al. ( 2012), and Alvarez et al. ( 2012). Whilst Chen et al. ( 2013) report enhanced N_2_O emissions (compared to unamended controls) at C/N ratios < 45, our results during the first 30 days of the incubation did not follow this pattern under both aerobic (WFPS 60 and 75%) and anaerobic (WFPS 90%) conditions. Only the HC treatment (C/N = 13 and relatively high levels of mineral N and labile C) consistently showed higher N_2_O production. This effect was not as evident during the second 30 days, when the addition of urea provided sufficient available N to support microbial decomposition of the OAs and promoted N_2_O production (greater than the unamended control) in all OAs except RB with high C/N ratio. Our data supports the hypothesis of Chen et al. ( 2013) that organic residues supply only a small fraction of the N requirement needed to promote mineralisation and N_2_O production.

Total N_2_O emissions from the clay soil were generally lower than from the sandy loam soil at all WFPS levels and OAs, at both 30 and 60 days. This is consistent with observations of Jarecki et al. ( 2008), Cayuela et al. ( 2010) and Wang et al. ( 2011) but in direct contrast to Chen et al. ( 2013). Whilst we agree with De Visscher et al. ( 1998) that soils with high CEC (e.g. clays) may facilitate immobilisation of NH_4_^+^ at cation exchange sites our mineral N data after 60 days does not support this hypothesis. The coarser textured sandy loam could be favouring N_2_O production through nitrification as observed by Cayuela et al. ( 2010), however there are significantly larger amounts of N_2_O being produced at 90% WFPS in the sandy loam which can only be attributed to denitrification. This suggests that the contrasting low N_2_O emissions observed from the clay soil at 90% WFPS are due to the conversion of N_2_O to N_2_. Our observation of higher levels of NO_3_^-^ in the clay soil after 60 days could potentially negate this conclusion, however the addition of urea at day 30, effectively removed any constraints on C and N mineralisation and NO_3_^-^ levels in both soils would still be relatively high even under conditions favouring denitrification.

Our study supports the weight of literature that biochar can significantly reduce N_2_O emissions in both aerobic and anaerobic situations. Our reductions in emissions during the second 30 days after fertilisation ranged from 42-64% and are of the same order of magnitude as those reported by Wang et al. ( 2011); Zhang et al. ( 2010, 2012) and van Zwieten et al. ( 2010, 2013). Lehmann et al. ( 2006) and van Zwieten et al. ( 2009) reported reduction in N_2_O emissions due to N immobilisation however we added additional mineral N and both NH_4_^+^ and NO_3_^+^ levels remained high in the RB treatment which confirms our earlier assertion that the highly resistant nature of high C/N rice husk biochar has little (if any) bioavailable C to fuel the denitrification process.

## Conclusions

This laboratory incubation provides critical information with respect to the practical utilisation of a range of OAs on the production of GHGs from soils of contrasting textures in response to changes in WFPS. The addition of OAs significantly increased CO_2_ production. Nitrous oxide emissions decreased on application of OAs, except in the case of the high N compost. In terms of CO_2_, regression models which included DM, C/N ratio, soil CEC and WFPS as input variables were able to describe CO_2_ production over 30 and 60 days, the latter after the application of urea (equivalent to 200 kg N ha^-1^). The model was not as effective when predicting CO_2_ emissions from composts with high levels of available (labile) C and (mineral) N. Regression models using the same input parameters as CO_2_ were able to explain the production of N_2_O at 30 and 60 days. The application of RB reduced N_2_O emissions (during the 30 days after urea was applied) by as much as 42-64% depending on WFPS. Reductions in both CO_2_ and N_2_O emissions after application of RB with high C/N ratio are due to a reduced bioavailability of C and not immobilisation of N. Further studies are needed to verify the mechanism in which RB reduces N_2_O emissions.

## References

[CR1] Ajwa HA, Tabatabai MA (1994). Decomposition of different organic materials in soils. Bio Fertil Soils.

[CR2] Alotaibi KD, Schoenau JJ (2013). Greenhouse gas emissions and nutrient supply rates in soil amended with biofuel production by-products. Bio Fertil Soils.

[CR3] Alvarez C, Costantini A, Alvarez CR, Alves BJR, Jantalia CP, Martellotto EE, Urquiaga S (2012). Soil nitrous oxide emissions under different management practices in the semiarid region of the Argentinian Pampas. Nutr Cycl Agroecosyt.

[CR4] Ambus P, Jensen ES, Roberton GP (2001). Nitrous oxide and N-leaching losses from agricultural soil: Influence of crop residue particle size, quality and placement. *Phyton*. Annales Rei Botanicae.

[CR5] Amiri MA, Fallahi E (2009). Impact of animal manure on soil chemistry, mineral nutrients, yield, and fruit quality in ‘golden delicious’ apple. Plant Nutr.

[CR6] Andersen AJ, Petersen SO (2009). Effects of C and N availability and soil-water potential interactions on N_2_O evolution and PLFA composition. Soil Biol Biochem.

[CR7] Aulakh MS, Rennie DA, Paul EA (1984). Gaseous nitrogen losses from soil under zero-till as compared with conventional-till management systems. Environ Qual.

[CR8] (2003). Composts, Soil Conditioners and Mulches.

[CR9] Barry GA, Rayment GE, Bloesch PM, Price A, Bruce RC, Johnstone M, Rayment GE (2000). Recycling Sugar Industry by-Products and Municipal Biosolids on Canelands.

[CR10] Bateman EJ, Baggs EM (2005). Contributions of nitrification and denitrification to N_2_O emissions from soils at different water-filled pore space. Biol Fertil Soils.

[CR11] Beare MH, Gregorich EG, St-Georges P (2009). Compaction effects on CO_2_ and N_2_O production during drying and rewetting of soil. Soil Biol Biochem.

[CR12] Biala J, Smeal C (2008). The Challenges of Implementing On-Site Composting in an Industrial Manufacturing Business.

[CR13] Bolan NS, Saggar S, Luo JF, Bhandral R, Singh J (2004). Gaseous emissions of nitrogen from grazed pastures: Processes, measurements, and modelling, environmental implications and mitigation. Adv Agron.

[CR14] Carter MR, Gregorich EG (2008). Soil Sampling and Methods of Analysis.

[CR15] Cavigelli M, Robertson G (2001). Role of denitrifier diversity in rates of nitrous oxide consumption in a terrestrial ecosystem. Soil Biol Biochem.

[CR16] Cayuela ML, Velthof GL, Mondini C, Sinicco T, Van Groenigen JW (2010). Nitrous oxide and carbon dioxide emissions during initial decomposition of animal by-products applied as fertilisers to soils. Geoderma.

[CR17] Chan KY, Zwieten LV, Meszaros I, Downie A, Joseph S (2008). Using poultry litter biochars as soil amendments. Soil Res.

[CR18] Chapman LS, Hunter HM, Eyles AG, Rayment GE (1996). Australian Sugar Industry by-products recycle plant nutrients. Downstream Effects of Land Use.

[CR19] Chatterjee A, Vance GF, Pendall E, Stahl PD (2008). Timber harvesting alters soil carbon mineralization and microbial community structure in coniferous forests. Soil Biol Biochem.

[CR20] Chen H, Li X, Hu F, Shi W (2013). Soil nitrous oxide emissions following crop residue addition: a meta-analysis. Glob Chang Biol.

[CR21] Craine J, Morrow C, Fierer N (2007). Microbial nitrogen limitation increases decomposition. Ecology.

[CR22] Dalal RC, Wang W, Robertson GP, Parton WJ (2003). Nitrous oxide emissions from Australian agricultural lands and mitigation options: a review. Soil Res.

[CR23] Davidson EA, Swank WT, Perry TO (1986). Distinguishing between nitrification and denitrification sources of gaseous nitrogen-production in soil. Appl Environ Microbiol.

[CR24] De Visscher A, Boeckx P, Van Cleempu O (1998). Interaction between nitrous oxide formation and methane oxidation in soils: Influence of cation exchange phenomena. Environ Qual.

[CR25] Drewitt GB, Black TA, Nesic Z, Humphreys ER, Jork EM, Swanson R, Ethier GJ, Griffis T, Morgenstern K (2002). Measuring forest floor CO_2_ fluxes in a Douglas-fir forest. Agr Forest Meteorol.

[CR26] Eichner MJ (1990). Nitrous oxide emissions from fertilized soil: summary of available data. Environ Qual.

[CR27] FAO-STAT 2014.http://faostat.fao.org/site/567/DesktopDefault.aspx?PageID=567#ancor

[CR28] Forster P, Ramaswamy V, Artaxo P, Berntsen T, Betts R, Fahey DW, Haywood J, Lean J, Lowe DC, Myhre G, Nganga J, Prinn R, Raga G, Schulz M, Dorland RV, Solomon S, Qin D, Manning M, Chen Z, Marquis M, Averyt KB, Tignor M, Miller HL (2007). Changes in Atmospheric Constituents and in Radiative Forcing. Climate Change 2007: The Physical Science Basis. Contribution of Working Group I to the Fourth Assessment Report of the Intergovernmental Panel on Climate Change.

[CR29] Frank AB, Liebig MA, Hanson JD (2002). Soil carbon dioxide fluxes in northern semiarid grasslands. Soil Biol Biochem.

[CR30] Garcia-Montiel DC, Melillo JM, Steudler PA, Cerri CC, Piccolo MC (2003). Carbon limitations to nitrous oxide emissions in a humid tropical forest of the Brazilian Amazon. Biol Fertil Soils.

[CR31] Haefele SM, Konboon Y, Wongboon W, Amarante S, Maarifat AA, Pfeiffer EM, Knoblauch C (2011). Effects and fate of biochar from rice residues in rice-based systems. Field Crops Res.

[CR32] Hartz TK, Mitchell JP, Giannini C (2000). Nitrogen and carbon mineralization dynamics of manures and composts. Hort Sci.

[CR33] Huang Y, Zou J, Zheng X, Wang Y, Xu X (2004). Nitrous oxide emissions as influenced by amendment of plant residues with different C:N ratios. Soil Biol Biochem.

[CR34] (1994). Radiative Forcing of Climate Change. The 1994 Report of the Scientific Assessment Working Group of IPCC. Summary for Policymakers.

[CR35] (2007). Climate Change 2007: Working Group I: The Physical Science Basis.

[CR36] Ippolito JA, Laird DA, Busscher WJ (2012). Environmental benefits of biochar. Environ Qual.

[CR37] Jarecki MK, Parkin TB, Chan ASK, Hatfield JL, Jones R (2008). Greenhouse gas emissions from two soils receiving nitrogen fertilizer and swine manure slurry. Environ Qual.

[CR38] Ji Y, Liu G, Ma J, Xu H, Yag K (2012). Effect of controlled-release fertilizer on nitrous oxide emission from a winter wheat field. Nutr Cycl Agroecosyt.

[CR39] Kroeze C, Mosie A, Bouwman L (1999). Closing global N_2_O budget: a retrospective analysis 1500–1994. Glob Biogeochem Cycles.

[CR40] Kumar S, Malik RS, Dahiya IS (1985). Influence of different organic wastes upon water retention, transmission and contact characteristics of a sandy soil. Soil Res.

[CR41] Law Y, Lant P, Yuan Z (2011). The effect of pH on N_2_O production under aerobic conditions in a partial nitritation system. Water Res.

[CR42] Lehmann J, Gaunt J, Rondon M (2006). Biochar sequestration in terrestrial ecosystems - a review. Mit Adapt Strat Glob Change.

[CR43] Li L-J, You M-Y, Shi H-A, Ding X-L, Qiao Y-F, Han X-Z (2013). Soil CO_2_ emissions from a cultivated Mollisol: Effects of organic amendments, soil temperature, and moisture. Eur J Soil Biol.

[CR44] Li X, Hu F, Shi W (2013). Plant material addition affects soil nitrous oxide production differently between aerobic and oxygen-limited conditions. Appl Soil Ecol.

[CR45] Liang B, Solomon D, Kinyangi J, Grossman J, O'Neil B, Skjemstad JO, Thies J, Luizao FJ, Petersen J, Neves EG (2006). Black carbon increases cation exchange capacity in soil. Soil Sci Soc Am J.

[CR46] Linn DM, Doran JW (1984). Effect of water-filled pore space on carbon dioxide and nitrous oxide production in tilled and nontilled soils. Soil Sci Soc Am J.

[CR47] Major J, Lehmann J, Rondon M, Goodale C (2010). Fate of soil-applied black carbon downward migration leaching and soil respiration. Glob Chang Biol.

[CR48] Masulili A, Utomo W, Syechfani M (2010). Rice husk biochar for rice based cropping system in acid soil 1. The characteristics of rice husk biochar and its influence on the properties of acid sulfate soils and rice growth in West Kalimantan, Indonesia. J Agric Sci.

[CR49] (2013). Wolfram Research.

[CR50] Meunchang S, Panichsakpatana S, Weaver RW (2005). Co-composting of filter cake and bagasse; by-products from a sugar mill. Bioresour Technol.

[CR51] Mohn J, Schürmann A, Hagedorn F, Schleppi P, Bachofen R (2000). Increased rates of denitrification in nitrogen-treated forests soils. For Ecol Manage.

[CR52] Naidu R, Syers JK (1992). Influence of sugarcane millmud, lime, and phosphorus, on soil chemical properties and the growth of *Leucaena leucocephala* in an Oxisol from Fiji. Bioresour Technol.

[CR53] Negro MJ, Solano ML, Caria P, Carrasco J (1999). Composting of sweet sorghum bagasse with other waste. Bioresour Technol.

[CR54] Nguyen DH, Biala J, Grace PR, Scheer C, Rowlings D, Bruce R (2013). Effects of rice husk biochar and sugar-mill by-products on methane consumption from two different soils. Proc Aust Soc Sugar Cane Technol.

[CR55] Nguyen DH, Grace PR, Scheer C, Rowlings D (2014). Determining gas sampling timelines for estimating emissions in small chamber incubation experiments. IOSR-JEN.

[CR56] Peng X, Ye LL, Wang CH, Zhou H, Sun B (2011). Temperature- and duration-dependent rice straw-derived biochar: Characteristics and its effects on soil properties of an Ultisol in southern China. Soil Till Res.

[CR57] Pérez CA, Carmona MR, Fariña JM, Armesto JJ (2010). Effects of nitrate and labile carbon on denitrification of southern temperate forest soils. Chil J Agric Res.

[CR58] Quilty JR, Cattle SR (2011). Use and understanding of organic amendments in Australian agriculture: A review. Soil Res.

[CR59] Ravishankara A, Daniel J, Portmann R (2009). Nitrous oxide (N_2_O): The dominant ozone-depleting substance emitted in the 21st century. Science.

[CR60] Regina K, Nykänen H, Maljanen M, Silvola J, Martikainen PJ (1998). Emissions of N_2_O and NO and net nitrogen mineralization in a boreal forested peatland treated with different nitrogen compounds. Can J For Res.

[CR61] Robertson GP, Tiedje JM (1988). Deforestation alters denitrification in a lowland tropical rain forest. Nature.

[CR62] Rogovska N, Laird D, Cruse R, Fleming P, Parkin T, Meek D (2011). Impact of biochar on manure carbon stabilization and greenhouse gas emissions. Soil Sci Soc Am J.

[CR63] Rudaz AO, Walti E, Kyburz G, Lehmann P, Fuhrer J (1999). Temporal variation in N_2_O and N_2_ fluxes from a permanent pasture in Switzerland in relation to management, soil water content and soil temperature. Agric Ecosyst Environ.

[CR64] Ruser R, Flessa H, Russow R, Schmidt G, Munch FBJC (2006). Emissions of N_2_O, N_2_ and CO_2_ from soil fertilized with nitrate: effect of compaction, soil moisture and rewetting. Soil Biol Biochem.

[CR65] Shiralipour A, McConnell DB, Smith WH (1992). Physical and chemical properties of soils as affected by municipal solid waste compost application. Biomass Bioenergy.

[CR66] Singh BP, Hatton B, Singh B, Cowie A, Kathuria A (2010). Influence of biochar on nitrous oxide emissions and nitrogen leaching from two contrasting soils. Environ Qual.

[CR67] Singh BP, Cowie AL, Smernik RJ (2012). Biochar carbon stability in a clayey soil s a function of feedstock and pyrolysis temperature. Environ Sci Technol.

[CR68] Speir TW, Horswell J, Van Schaik AP, McLaren RG, Fietje G (2004). Composted biosolids enhance fertility of a sandy loam soil under dairy pasture. Bio Fertil Soils.

[CR69] Tans P (2012). Monthly mean Concentration CO2 at the Mauna Loa Observatory.

[CR70] Uchida Y, Rein I, Akiyama H, Yagi K (2013). Contribution of nitrification and denitrification to nitrous oxide emissions in Andosol and from Fluvisol after coated urea application. Soil Sci Plant Nutr.

[CR71] Umamaheswaran K, Batra VS (2008). Physico-chemical characterisation of Indian biomass ashes. Fuel.

[CR72] Van Zwieten L, Singh B, Joseph S, Kimber S, Cowie A, Chan K, Lehmann J, Joseph S (2009). Biochar and Emissions of Non-CO_2_ Greenhouse Gases from Soil. Biochar for Environmental Management: Science and Technology.

[CR73] Van Zwieten L, Kimber S, Morris S, Downie A, Berger E, Rust J (2010). Influence of biochar on flux of N_2_O and CO_2_ from Ferrosol. Soil Res.

[CR74] Van Zwieten L, Kimber SWL, Morris SG, Singh BP, Grace PR, Scheer C, Rust J, Downie A, Cowie AL (2013). Pyrolysing poultry litter reduces N2O and CO2 fluxes. Sci Total Environ.

[CR75] Verheijen F, Jeffery S, Bastos AC, van der Velde M, Diafas I (2009). Biochar Application to Soil - A Critical Scientific review of Effects on Soil Properties, Processes and Functions. EUR 24099 EN.

[CR76] Virgil MF, Kissel DE (1991). Equations for estimating the amount of nitrogen mineralized from crop residues. Soil Sci Soc Am J.

[CR77] Wallenstein MD, Peterjohn WT, Schlesinger WN (2006). Fertilization effects on denitrification and N cycling in an aggrading forest. Ecol Appl.

[CR78] Wang J, Zhang M, Xiong Z, Liu P, Pan G (2011). Effects of biochar addition on N_2_O and CO_2_ emissions from two paddy soils. Biol Fertil Soils.

[CR79] Wang J, Pan X, Liu Y, Zhang X, Xiong Z (2012). Effects of biochar amendment in two soils on greenhouse gas emissions and crop production. Plant Soil.

[CR80] Weier KL, Doran JW, Power JF, Walters DT (1993). Denitrification and the dinitrogen/nitrous oxide ratio as affected by soil water, available carbon and nitrate. Soil Sci Soc Am J.

[CR81] Whalen J, Chang C, Clayton G, Carefoot J (2000). Cattle manure amendments can increase the pH of acid soils. Soil Sci Soc Am J.

[CR82] Widowati, Utomo WH, Soehono LA, Guritno B (2011). Effect of biochar on the release and loss of nitrogen from urea fertilization. J Agric Food Tech.

[CR83] Yaduvanshi NPS, Yadav DV (1999). Effects of sulphitation press mud and nitrogen fertilizer on biomass, nitrogen economy and plant composition in sugarcane and on soil chemical properties. J Agric Sci.

[CR84] Yuan JH, Xu RK, Wang N, Li JY (2011). Amendment of acid soils with crop residues and biochars. Pedosphere.

[CR85] Zebarth BJ, Neilsen JH, Hogue E, Neilsen D (1999). Influence of organic waste amendment on selected soil physical and chemical properties. Can J Soil Sci.

[CR86] Zhang AF, Cui LQ, Pan GX, Li LQ, Hussain Q, Zhang XH, Zheng JW, Crowley D (2010). Effect of biochar amendment on yield and methane and nitrous oxide emissions from a rice paddy from Tai Lake plain, China. Agric Ecosyst Environ.

[CR87] Zhang A, Bian R, Pan G, Cui L, Hussain Q, Li L, Zheng J, Zheng J, Zhang X, Han X, Yu X (2012). Effects of biochar amendment on soil quality, crop yield and greenhouse gas emission in a Chinese rice paddy: A field study of 2 consecutive rice growing cycles. Field Crops Res.

[CR88] Zhao Y, Wang P, Li J, Chen Y, Ying X, Liu S (2009). The effects of two organic manures on soil properties and crop yields on a temperate calcareous soil under a wheat–maize cropping system. Eur J Agron.

[CR89] Zheng J, Stewart C, Cotrufo MF (2012). Biochar and nitrogen fertilizer alters soil nitrogen dynamics and greenhouse gas fluxes from two temperate soils. Environ Qual.

[CR90] Zimmerman AR, Gao M, Ahn MY (2011). Positive and negative carbon mineralization priming effects among a variety of biochar-amended soils. Soil Biol Biochem.

